# Computational Visual Stress Level Analysis of Calcareous Algae Exposed to Sedimentation

**DOI:** 10.1371/journal.pone.0157329

**Published:** 2016-06-10

**Authors:** Jonas Osterloff, Ingunn Nilssen, Ingvar Eide, Marcia Abreu de Oliveira Figueiredo, Frederico Tapajós de Souza Tâmega, Tim W. Nattkemper

**Affiliations:** 1 Biodata Mining Group, Faculty of Technology, Bielefeld University, Bielefeld, Germany; 2 Statoil ASA, Research and Technology, N-7005 Trondheim, Norway; 3 Trondhjem Biological Station, Department of Biology, Norwegian University of Science and Technology, N-7491 Trondheim, Norway; 4 Instituto de Pesquisa Jardim Botanico do Rio de Janeiro, CEP 22.460-030, Rio de Janeiro, RJ, Brazil; 5 Instituto Biodiversidade Marinha, 22.793-000, Rio de Janeiro, RJ, Brazil; 6 Instituto de Estudos do Mar Almirante Paulo Moreira, 28.930-000, Arraial do Cabo, RJ, Brazil; Northwest Fisheries Science Center, NOAA Fisheries, UNITED STATES

## Abstract

This paper presents a machine learning based approach for analyses of photos collected from laboratory experiments conducted to assess the potential impact of water-based drill cuttings on deep-water rhodolith-forming calcareous algae. This pilot study uses imaging technology to quantify and monitor the stress levels of the calcareous algae *Mesophyllum engelhartii* (Foslie) Adey caused by various degrees of light exposure, flow intensity and amount of sediment. A machine learning based algorithm was applied to assess the temporal variation of the calcareous algae size (∼ mass) and color automatically. Measured size and color were correlated to the photosynthetic efficiency (maximum quantum yield of charge separation in photosystem II, $\Phi_{{\text{PSII}}_{\text{max}}}$) and degree of sediment coverage using multivariate regression. The multivariate regression showed correlations between time and calcareous algae sizes, as well as correlations between fluorescence and calcareous algae colors.

## Introduction

Increasing anthropogenic activities in marine areas call for a holistic environmental monitoring approach using new and more sophisticated models and sensor systems that enable predictions and measurement of impact on organisms of interest [1]. One important input to modeling is knowledge generated through laboratory experiments, determining various levels of stress on the organisms of interest. A common way to measure stress is through visual documentation of behavioral changes, change in abundance [2–4], change of the spectral response [5] or change of the visual appearance [6, 7] of organisms.

The investigated calcareous algae species, *Mesophyllum engelhartii* (Foslie) Adey, belongs to the *Phylum Rhodophyta* under the Order *Hapalidiales*. Calcareous algae play an important ecological role in coastal habitats [8–10] contributing to the formation of rhodoliths. Rhodoliths are made from dead calcareous algae and other calcifying organisms [11]. These multi-spherical structures are creating a habitat for other organisms living on, between and in the structures [12]. Calcareous algae are found down to about 250 meters water depth [13–15] and the largest occurrence of rhodolith beds are found in the southwest Atlantic on the Brazilian continental shelf [16–18]. These algae communities may be disturbed by natural sedimentation and/or sedimentation from anthropogenic activities such as fish-trawling, mining [19] and discharges of drill cuttings from oil and gas drilling activities [20].

In a previous study the impact from sedimentation of drill cuttings on live calcareous algae was studied by measuring sediment coverage (*SC*) and photosynthetic efficiency (*P*) [6]. The study was part of the Peregrino Environmental Monitoring Calcareous Algae Project (PEMCA). Rhodoliths, partly covered with calcareous algae, were collected from the Peregrino oil field off the coast of Brazil. The impact of sedimentation was investigated in a laboratory flow-through system, varying light exposure (L), flow rate (F), amount of sediment (S) and time (T). Photosynthetic efficiency (*P*) was measured as maximum quantum yield of charge separation in photosystem II, $\Phi_{{\text{PSII}}_{\text{max}}}$. These results have been published elsewhere [6].

The present paper describes a new method to extend the observed impact variables (SC, P) by measurements of size and color of calcareous algae in digital photos recorded during the experiments.

Using photos for environmental monitoring generates a large number of images implying that a manual evaluation is extremely demanding regarding time and resources and requires a careful and professional execution, as for instance outlined in [21].

To support the evaluation and labeling of the photos performed by marine biology experts different software tools have been proposed such as Coral Point Count with Excel extensions (CPCe) [22]. Recently, web-based image annotation and labeling systems have been proposed to support web-based sharing of photos and collaboration, such as BIIGLE (Benthic Image Indexing, Graphical Labeling and Exploration) [23], which was successfully applied in [4, 24–28], and CoralNet [29], the latter one providing for instance an automated point classification for corals as well.

Since humans have a limited capability to quantify visual features, such as color change, in an objective way, we propose a machine learning based approach to compute size and color of the calcareous algae from photos recorded at different time points. A machine learning algorithm is trained with a number of manual image annotations. The trained classifier is applied for the full automatic segmentation of the calcareous algae, enabling the quantification of the calcareous algae size and color over time. To learn the classification function a H^2^SOM (Hierarchical Hyperbolic Self-Organizing Map) [30] algorithm was applied. This unsupervised learning algorithm has previously been used for cold-water coral segmentation in ROV video frames [24] and for poly-metallic nodule segmentation [31, 32]. Nevertheless, the integration of the basic algorithm into an image analysis pipeline needed to be modified substantially regarding pre-processing, feature computation, pixel classification function and post-processing.

**Fig 1:**
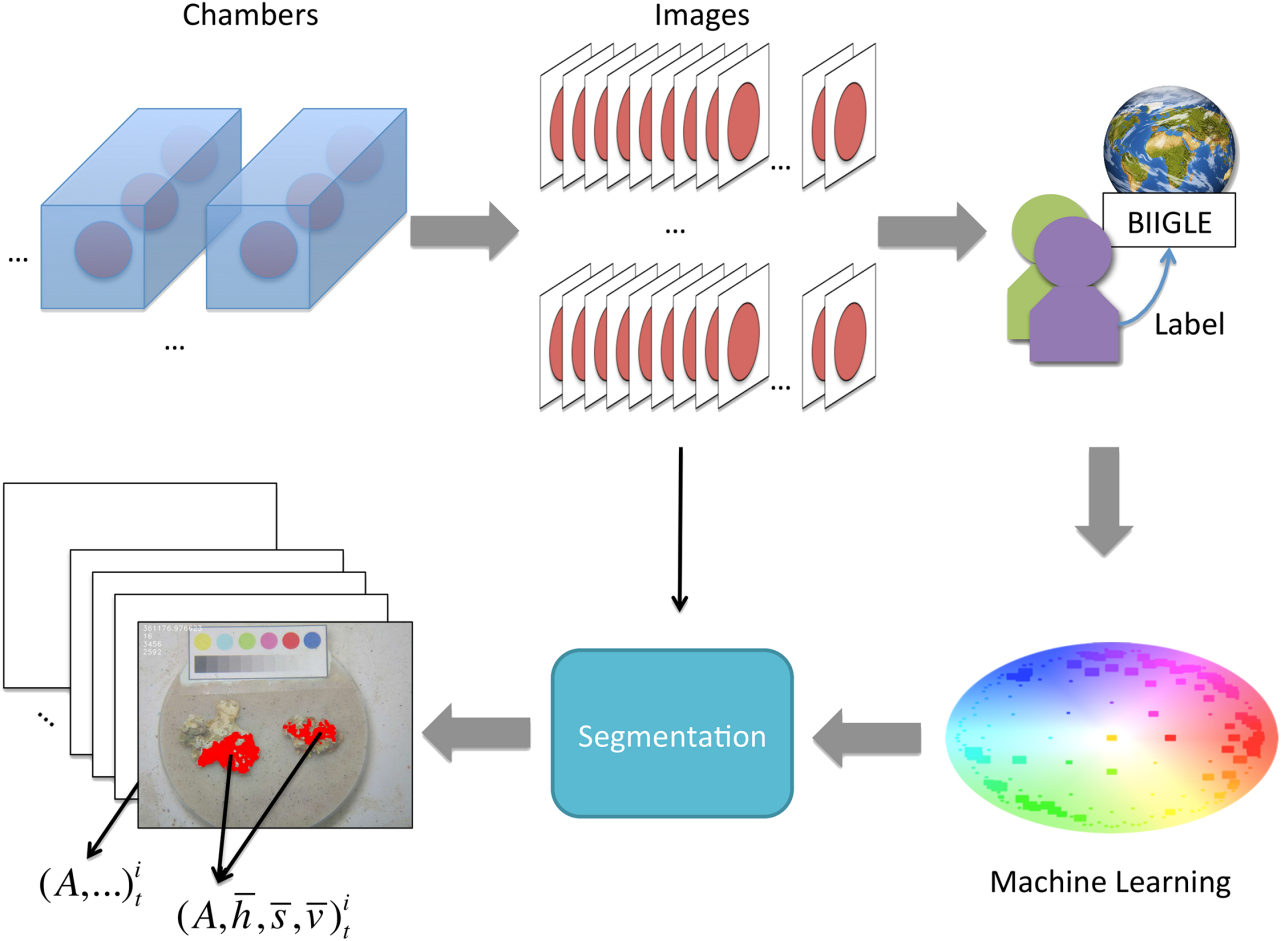
A simplified illustration of the impact quantification process. The process is starting on the upper left with the laboratory experiment, continuing with the image recording, the labeling by the experts, the application of the unsupervised machine learning algorithm (see Machine Learning section for details) and ending with the image segmentation and the measuring of size and color of the segmented area.

A simplified overview of the whole impact quantification process is given in Fig 1.

## Material

Samples of live calcareous algae *Mesophyllum engelhartii* (Foslie) Adey were collected from 94–103m water depth at the Peregrino oil production field (23°13′28.34′′S, 41°50′55.73′′W) located off the Brazilian Atlantic coast [11]. Light intensity (L), flow rate (F) and sediment amount (S) could be varied independently as described in [6]. In order to optimize the experiment, the three predictor variables (L, F, S) were combined according to a Central Composite Face (CCF) design with center point [33]. This enables regression modeling to describe linear and non-linear relationships and interactions between the variables. The full factorial design gives 15 combinations of L, F, and S. Two of these combinations were duplicated and two were triplicated (i.e. technical replicates). Using the 15 combinations and technical replicates resulted in a total of 21 experiments which were carried out in 21 chambers (Table 1). In addition controls were kept in complete darkness and without sediment. The area of the chamber bottom was approximately 0.06m^2^ and the height of was 0.1 m resulting in a volume of approximately 6 L. The light levels (L) used in the exposure studies were 3, 6.6, or 10 μmol m^−2^ s^−1^, the latter value corresponding to measured light conditions at the sea floor on the Peregrino field. The flow rates (F) used were 0.04, 0.07, or 0.09 m s^−1^. The amounts of sediment (S), applied at the beginning of the experiment (T0), were 600, 900, or 1200 g per chamber. The chosen amounts resulted in a sediment coverage (*SC*) of the calcareous algae ranging from uncovered to completely covered, implying that also non- and very low sediment covered calcareous algae are included in the analysis. Six parallels (i.e. biological replicates) of rhodoliths covered with healthy calcareous algae were placed in each chamber, so each of the 21 experiments were conducted with six samples.

**Table 1:** Chamber configurations for the CCF design.

Chamber	$L[\frac{\mu \text{mol}}{{\text{m}}^{2}s}]$	$F[\frac{\text{m}}{\text{s}}]$	S[g]
1	3.0	0.04	600
2	10.0	0.09	600
3	3.0	0.09	600
4	10.0	0.04	600
5	3.0	0.04	1200
6	10.0	0.04	1200
7	3.0	0.09	1200
8	10.0	0.09	1200
9	3.0	0.07	900
10	3.0	0.07	900
11	10.0	0.07	900
12	6.6	0.04	900
13	6.6	0.04	900
14	6.6	0.04	900
15	6.6	0.09	900
16	6.6	0.09	900
17	6.6	0.09	900
18	6.6	0.07	600
19	6.6	0.07	1200
20	6.6	0.07	900
21	6.6	0.07	900

The configurations for the 21 chambers for light intensity (L), flow rate (F), and sediment amount (S) are displayed. Replicated configurations are grouped.

**Fig 2:**
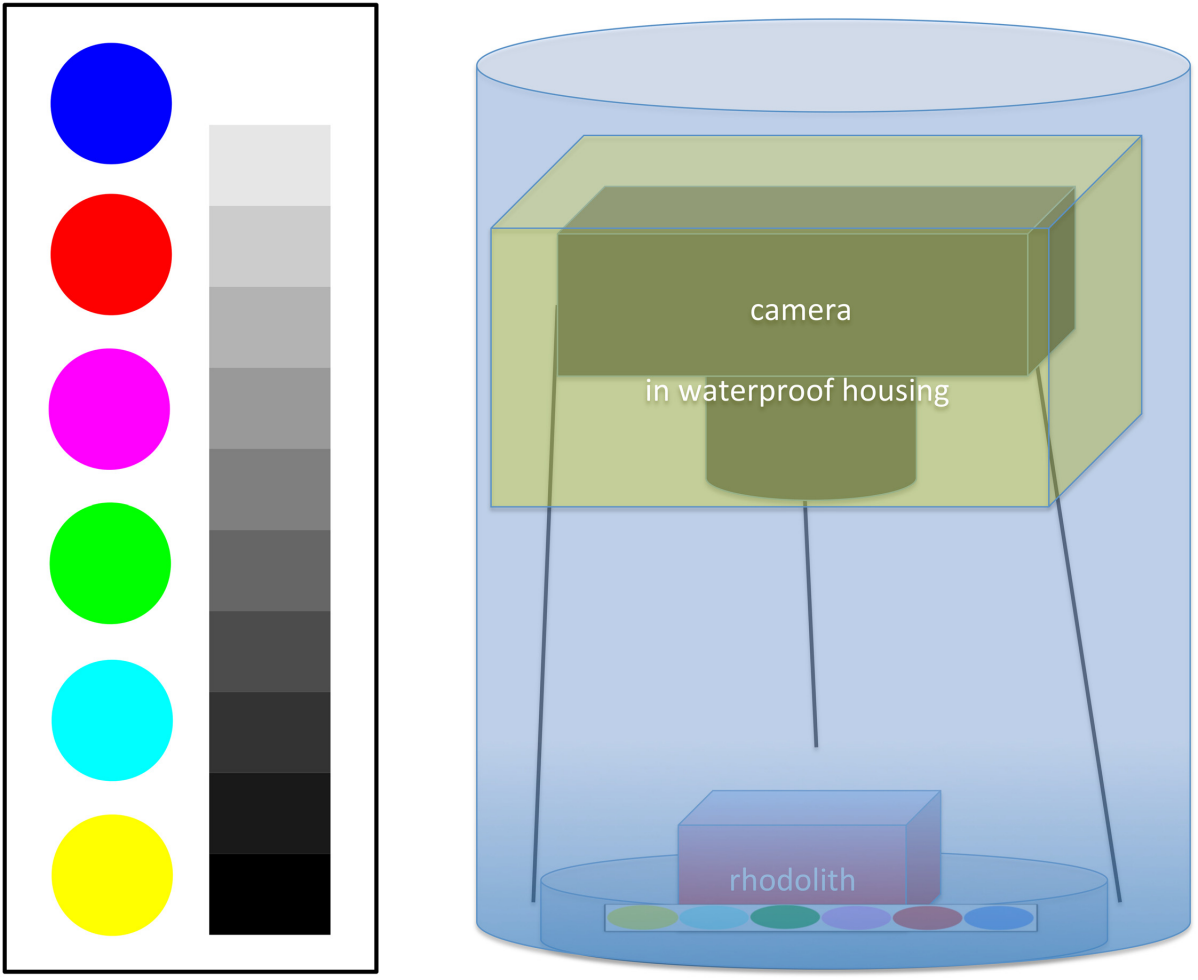
Illustration of color reference plate and camera setup. On the left the original color reference plate, used to obtain color constancy within the experiment, is displayed. Colors are referring to the RGB triplets (top-down): (0,0,255), (255,0,0), (255,0,255), (0,255,0), (0,255,255), (255,255,0). On the right a schematic layout of the underwater camera setup is presented, consisting of a water filled cylinder containing the sample, the color reference plate and the camera in the waterproof housing fixed on a tripod.

**Fig 3:**
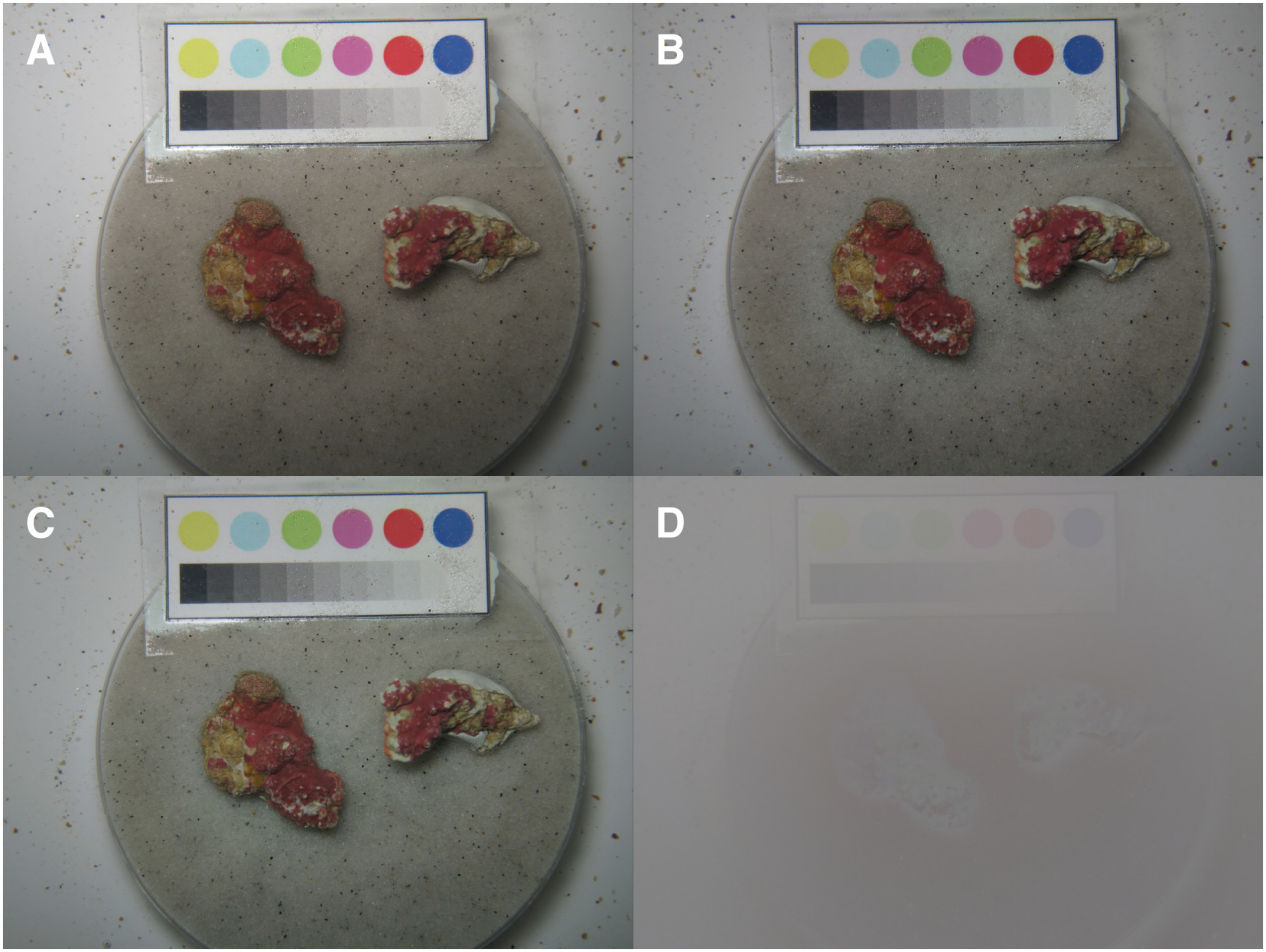
Example of an image recorded during the experiment completion and its different processing results of the pre-processing. The original image (A), the result of the illumination correction (B), the result of the color correction (C) and the difference image (D) are displayed. In the original image (A) the color reference plate is located in the upper part, providing input for the pre-processing. The two calcareous algae samples are located below the reference plate. Comparing the result image from the illumination correction (see Illumination Correction section for details) with the original image a slight decrease in brightness can be noticed. Due to the stable illumination conditions during image recording only little differences between the images can be recognized visually. The color correction (see Reference Plate Detection, Color and Zoom Correction section for details) is changing the colors very little as only minor color shifts appear. Most of the visible differences in the difference image are caused by the illumination correction.

In addition to the measurements reported in [6], photos of the calcareous algae samples were taken for purpose of the present paper during the weekly examination. For image-acquisition, the calcareous algae rodoliths were removed from their chambers and manually placed in a transparent, water-filled cylinder together with a color reference plate (Figs 2 and 3A). The reference plate, essential for a comparative study of the color over time, can be seen in the upper part of the images (Fig 3 upper left). The photos were taken with a Canon PowerShot G10, which was placed on a tripod to control the distance (15 cm) between camera and samples. The camera was in automatic mode with macro function and internal flash on. To eliminate light refractions and reflections samples and camera, the latter protected by a waterproof housing, were placed underwater inside the cylinder (Fig 2). The digital images had a resolution of 3456 × 2592 pixels. Each week 63 images (21 chambers × 3 images, 2 samples per photo), were taken summing up to *N* = 630 images for the whole experiment (T0, …, T9).

**Fig 4:**
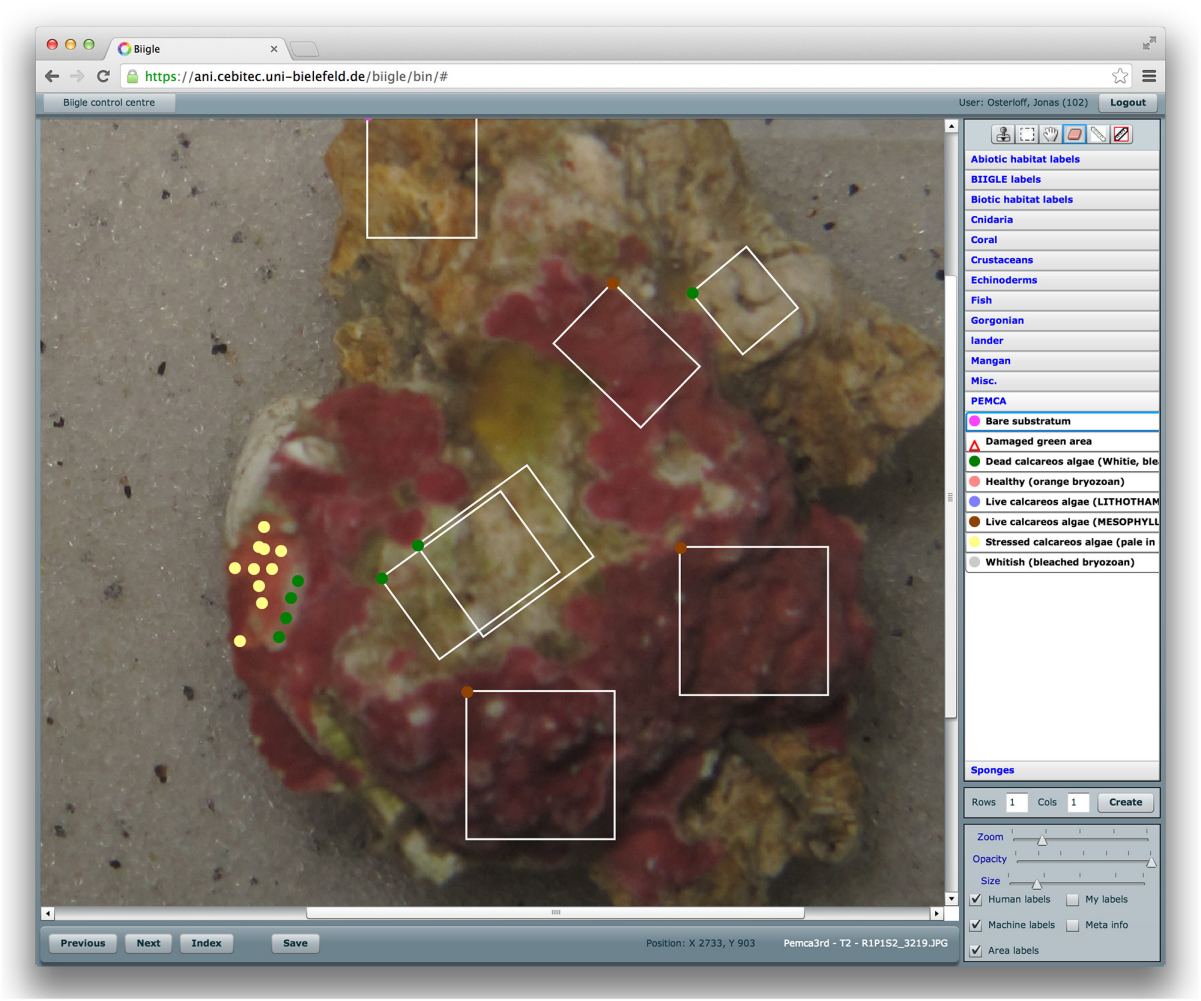
A screenshot of the BIIGLE system in a web-browser. The images of the calcareous algae were examined and labeled using the BIIGLE system. Images can also be zoomed to examine more details. The experts were allowed to select single point label or customizable frame labels. The single point labels are represented as filled colored circles, the customizable frame labels are represented as white outlined rectangles with a filled colored circle in the upper left corner of the individual rectangle. The colors of the filled circles indicate the class of the individual label. Red is representing “live” calcareous algae, yellow “stressed” calcareous algae, green “dead” calcareous algae and Pink “bare substratum”.

In order to collect a set of image annotations for training and testing the machine learning algorithm, the recorded images were uploaded to BIIGLE. With this online image annotation tool, biological experts from the Instituto Biodiversidade Marinha were manually labeling photo regions (pixel) as “live” calcareous algae (2404 labels), “stressed” calcareous algae (2834 labels), “dead” calcareous algae (4358 labels) and “bare substratum” (43 labels) on all *N* = 630 images. For the annotation, the experts were allowed to select single point label or customizable frame labels (Fig 4). These labels provided examples for the later machine learning training step (see Machine Learning section below). The labeling strategy was chosen to reduce the time needed for the labeling and simultaneously cover the whole variation between and within each label category over time. The “live” and “stressed” calcareous algae labels represent the so called “positive” class in the segmentation and the “dead”- and “bare substratum”-labels represent the so called “negative” class. All images and labels can be accessed via BIIGLE (https://ani.cebitec.uni-bielefeld.de/biigle/). To browse the data, a login with username **pemca** and password **pemca** is required. The data is stored in the project **Pemca3rd**, with the transects *T*0, …, *T*9. Image material is also published under DOI 10.4119/unibi/2775316.

## Methods

The permission to collect the calcareous algae samples for the experimental study described in the Material section was granted by the Chico Mendes Institute for Biodiversity Conservation SISBIO license n 20820-2 and 20826-1. The experimental study did not involve endangered or protected species.

The basic idea of the computational calcareous algae segmentation is to classify each pixel in each image to be i)“live” or “stressed” calcareous algae or ii) other, including “dead” calcareous algae. A pixel classifier is trained using data collected from the annotations provided by the experts (see Material section). From the pixel-wise classification, the regions of “live”/“stressed” calcareous algae are determined for each sample at each time point. The results are analyzed in combination with the previously measured parameters photosynthetic efficiency (*P*) and sediment coverage (*SC*) using multivariate analysis. The individual steps for image processing, pixel classification and final data analysis are described in detail in the following sections.

### Pre-Processing

Before the feature extraction and the machine learning can be applied, the recorded images need to be pre-processed.

#### Illumination Correction

In order to compensate illumination fluctuations in the images the *local space average color scaling* [34] is used on each image individually. The local space average color image is computed using a Gaussian blurring with *σ* = 0.093 ⋅ max(*I*_height_, *I*_width_). As the images were captured in sRGB [35], which assumes a gamma correction of 1/2.2, the images are gamma corrected and then divided pixel- and channel-wise (*c*) by the corresponding Gaussian filtered image *G*^(*n*)^:

$${\hat{I}}_{x,y,c}^{\left(n\right)}={\left(\frac{{\left(I_{x,y,c}^{\left(n\right)}\right)}^{2.2}}{\tau \cdot G_{x,y,c}^{\left(n\right)}}\right)}^{1/2.2}$$ (1)

*τ* is a scaling factor and set to *τ* = 2 in the whole study. An example for an illumination corrected image is displayed in Fig 3 (upper right).

#### Reference Plate Detection, Color and Zoom Correction

The first step in the color correction is the detection of the color circles in the reference plate using a Hough circle transformation [36]. The Hough transform computes a list of circle candidates and those that show a low color variance inside are selected taking the spatial layout of the color plate into account as well. The identified six color circles *C*_*l*_ (*l* = 1, …, 6) of each ${\hat{I}}^{\left(n\right)}$ are then used to calculate one RGB color triplet $c_{l}^{\left(n\right)}$ for each circle over all its pixels $p_{(x,y),c}^{\left(n\right)}$.

$$c_{l}^{\left(n\right)}=\left(\begin{array}{c} \underset{\left(x,y\right)\in {\text{circle}}_{l}}{\text{median}}\left(p_{(x,y),\text{red}}^{\left(n\right)}\right)\\ \underset{\left(x,y\right)\in {\text{circle}}_{l}}{\text{median}}\left(p_{(x,y),\text{green}}^{\left(n\right)}\right)\\ \underset{\left(x,y\right)\in {\text{circle}}_{l}}{\text{median}}\left(p_{(x,y),\text{blue}}^{\left(n\right)}\right) \end{array}\right)$$ (2)

**Fig 5:**
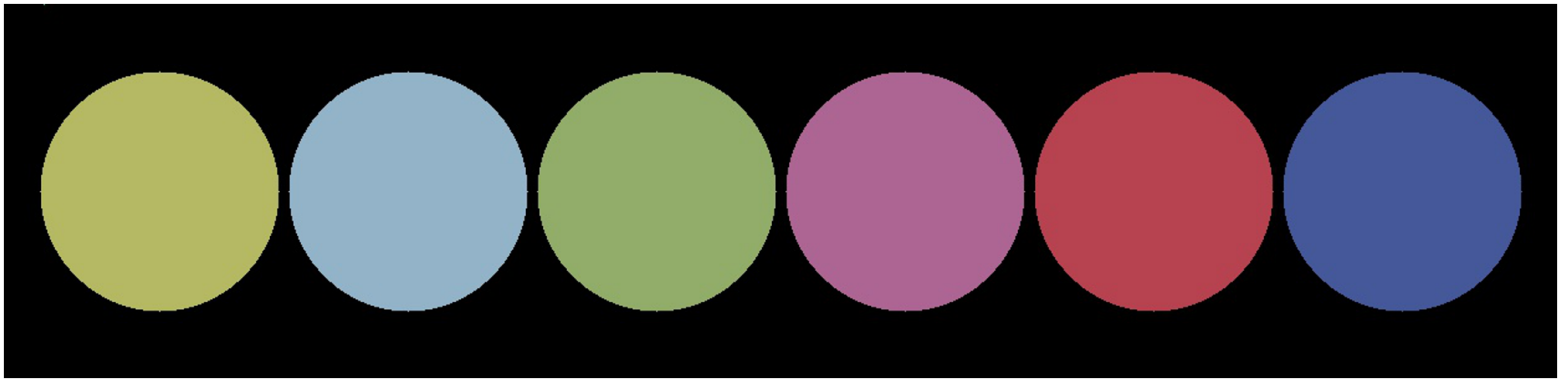
The average color reference plate. This reference is computed by averaging over the detected reference plates from all images used in this study (see text for details). Colors are referring the RGB triplets (left to right) (181,185,100), (147,179,200), (146,173,106), (173,101,147), (183,67,80), and (69,88,154).

In addition, overall median color references are computed for each of the six color circles (Eq 3) of all images (Fig 5).

$$\bar{c_{l}}=\left(\begin{array}{c} \underset{n}{\text{median}}\left(c_{l,\text{red}}^{\left(n\right)}\right)\\ \underset{n}{\text{median}}\left(c_{l,\text{green}}^{\left(n\right)}\right)\\ \underset{n}{\text{median}}\left(c_{l,\text{blue}}^{\left(n\right)}\right) \end{array}\right)$$ (3)

For each image ${\hat{I}}^{\left(n\right)}$ and color channel *c* ∈ {red, green, blue}, the individual, channel-wise gamma correction is calculated using the color RGB triplet $c_{l}^{\left(n\right)}$ (Eq 2) of the individual image and the overall median color references (Eq 3).

$$\gamma_{c}^{\left(n\right)}=\underset{l}{\text{median}}\left(\frac{\text{log}\left(\frac{c_{l,\text{red}}^{\left(n\right)}}{255}\right)}{\text{log}\left(\frac{\bar{c_{l,c}}}{255}\right)}\right)$$ (4)

This image specific gamma correction is applied channel-wise to all the images resulting in a new set of color corrected images (Fig 3 lower left)

$$J_{(x,y),c}^{\left(n\right)}={\left({\hat{I}}_{(x,y),c}^{\left(n\right)}\right)}^{\gamma_{c}^{\left(n\right)}}.$$ (5)

A difference image (${}^{\left(I_{x,y,c}^{\left(n\right)}\right)-\left(J_{x,y,c}^{\left(n\right)}\right)}{/}_{2}+128$) is presented in Fig 3 (lower right) to demonstrate the impact of the described illumination and color correction.

The layout of the reference plate is also used to correct zoom variations, which occurred for a significant number of images. The average distance $\bar{d}$ between the neighboring color circles (e.g. green and magenta) on each plate is evaluated from the five Euclidian circle distances *d*_*i*, *i*+1_

$$\bar{d}=\frac{1}{N}\sum_{n}\frac{1}{5}\sum_{i=1}^{5}d_{\left(i,i+1\right)}^{\left(n\right)}.$$ (6)

The difference between the average distance and the observed individual distances in image {*J*^(*n*)^} is used to rescale each image individually.

### Segmentation

Unsupervised learning is applied to learn a vector quantization of the image feature space (see Machine Learning section). Vector quantization fits a set of so called prototype vectors iteratively to a data set of feature vectors from the images describing pixel features. After the training step is finished, each prototype represents a group of pixels sharing similar features, i.e. a dense cloud in the feature space. The prototypes are assigned to the classes “live / stressed calcareous algae” and “other”, including “dead calcareous algae” and “bare substratum”, and are applied to classify all image pixel (see Prototype Label Mapping section). For the final segmentation result the classified image is post-processed (see Post Processing section).

#### Feature Extraction

One important step in vector quantization-based image segmentation is the selection of appropriate features and the collection of a training set. To collect a training set for the vector quantization learning, a selection of image pixels {*p*_*i*_} from a subset of all *N* images are chosen and their feature vectors {**x**^(*i*)^} are computed. Here, Median RGB values extracted on a 11 × 11 pixel neighborhood $(\eta_{11}^{\left(i\right)})$ for each color channel, are used as features since we observed such a representation to be sufficient for the initial task of calcareous algae segmentation by pixel classification.

$$x^{\left(i\right)}=\left(\begin{array}{c} \underset{\left(x,y\right)\in \eta_{11}^{\left(i\right)}}{\text{median}}\left(p_{(x,y),\text{red}}\right)\\ \underset{\left(x,y\right)\in \eta_{11}^{\left(i\right)}}{\text{median}}\left(p_{(x,y),\text{green}}\right)\\ \underset{\left(x,y\right)\in \eta_{11}^{\left(i\right)}}{\text{median}}\left(p_{(x,y),\text{blue}}\right) \end{array}\right)$$ (7)

To reduce the computation time, features are only extracted for pixels in a 2 × 2 grid resolution collecting a training set of about 2.5 million feature vectors {**x**^(*i*)^}.

#### Machine Learning

To perform vector quantization on the training data the unsupervised machine learning algorithm H^2^SOM [30] is applied.

H^2^SOMs with different parameter settings, like the number of prototypes, were trained and the best parameter set was determined using a customized criterion described in (S1 Text). A training set of feature vectors generated from 12.5% of the pixels chosen from a small subset (2%) of the entire image collection is used to construct the H^2^SOM with the best segmentation results. The H^2^SOM is trained using 30 × |{**x**^(*i*)^}| iterations, an exponentially decreasing learning rate of *α* = [0.99, 0.1], an eight-neighbor topology and three rings and therefore is consisting of 161 prototypes.

#### Prototype Label Mapping

The next step is to build a pixel classifier out of the trained H^2^SOM, using the feature vectors **x**^(*p*)^ of all images *J*^(*n*)^. We distinguish between feature vectors **x**^(*i*)^ used for the H^2^SOM training and feature vectors **x**^(*p*)^ from all images, but of course both are computed as described in Eq 7. The expert labels “live” and “stressed” from BIIGLE are used to build the classifier as follows. In general, we have observed that a differentiation between the categories “live” and “stressed” is problematic regarding the reproducibility of the results of the visual assessment. As a consequence a discrimination between these two categories has been neglected. The fused category is referred to as “live calcareous algae”.

First, a label *y*_*p*_ is assigned to each feature vector **x**^(*p*)^

$$y_{p}=\left\{\begin{array}{cc} 1, & \text{if}\;\text{the}\;\text{pixel}\;\text{was}\;\text{labeled}\;\text{as}\;“\text{live}”/“\text{stressed}”\;\text{calcareous}\;\text{algae}\\ 0, & \text{else} \end{array}\right.$$ (8)

Second, each feature vector **x**^(*p*)^ is assigned to one of the H^2^SOM prototypes {**u**^(*j*)^} (*j* = 1..161) that represents the features of **x**^(*p*)^ in the best possible way. This prototype is selected by the so called Best Matching Unit (BMU) criterion, that selects the prototype with the minimal Euclidian distance to **x**^(*p*)^ in the feature space. Next, all cluster prototypes are investigated in order to classify them. The prototypes constitute a Voronoi tessellation of the feature space

$$V_{j}=\left\{x^{\left(p\right)}|j=\underset{j^{\prime }}{\text{argmin}}\left\{\text{euclid}\left(u^{\left(j^{\prime }\right)},x^{\left(p\right)}\right)\right\}\right\}$$ (9)

with *q*_*j*_ = |*V*_*j*_| as the number of feature vectors that have been assigned to **u**^(*j*)^ and therefore are located in *V*_*j*_. In each Voronoi cell *V*_*j*_ we now analyze how many feature vectors stem from pixel, labelled as “live” or “stressed”:

$$V_{j}^{+}=\left\{x^{\left(p\right)}|j=\underset{j^{\prime }}{\text{argmin}}\left\{\text{euclid}\left(u^{\left(j^{\prime }\right)},x^{\left(p\right)}\right)\right\}\wedge y_{p}=1\right\},$$ (10)

with $q_{j}^{+}=\left|V_{j}^{+}\right|$ as the number of feature vectors in *V*_*j*_ that are labeled as “live”/“stressed” calcareous algae. The relation $s_{j}=q_{j}^{+}/q_{j}$ quantifies how well a prototype **u**^(*j*)^ resembles the features of live calcareous algae. Since we do not expect all **x**^(*p*)^ with *y*_*p*_ = 1 to be perfect representatives, only prototypes **u**^(*j*)^ with a high *s*_*j*_ will represent the positive class (presence of live calcareous algae).

Next the relation values {*s*_*j*_} are sorted in descending order:

$$s_{j}\to s_{l}\text{,}\;\text{with}\;s_{l}>s_{l-1}\forall l.$$ (11)

From this list, the first *L* prototypes *u*^(*l*)^ are chosen as a model for typical live calcareous algae color features. *L* is chosen so at least 80% of the entire positive training data (i.e. *y*_*i*_ = 1) is covered. This set of the first 16 “most live calcareous algae-like” prototypes is used for the pixel classification based segmentation in all images. In each image, each pixel *p* is mapped to each feature vector **x**^(*p*)^. The BMU of the feature vector is determined and if this unit is a live calcareous algae-like prototype, as described above, the pixel is labeled as live calcareous algae (*L*(**x**^(*p*)^) = 1) or otherwise as background (*L*(**x**^(*p*)^) = 0).

#### Post Processing

By classifying all feature vectors **x**^(*p*)^, binary images are created showing white pixels on live calcareous algae classified positions and black pixels otherwise. These binary images are post-processed with an opening filter mask [37] of a size 10 × 10 pixels to remove small areas of false positive classifications. The post-processed binary images represent the final segmentation result.

### Multivariate Data Analysis

**Fig 6:**
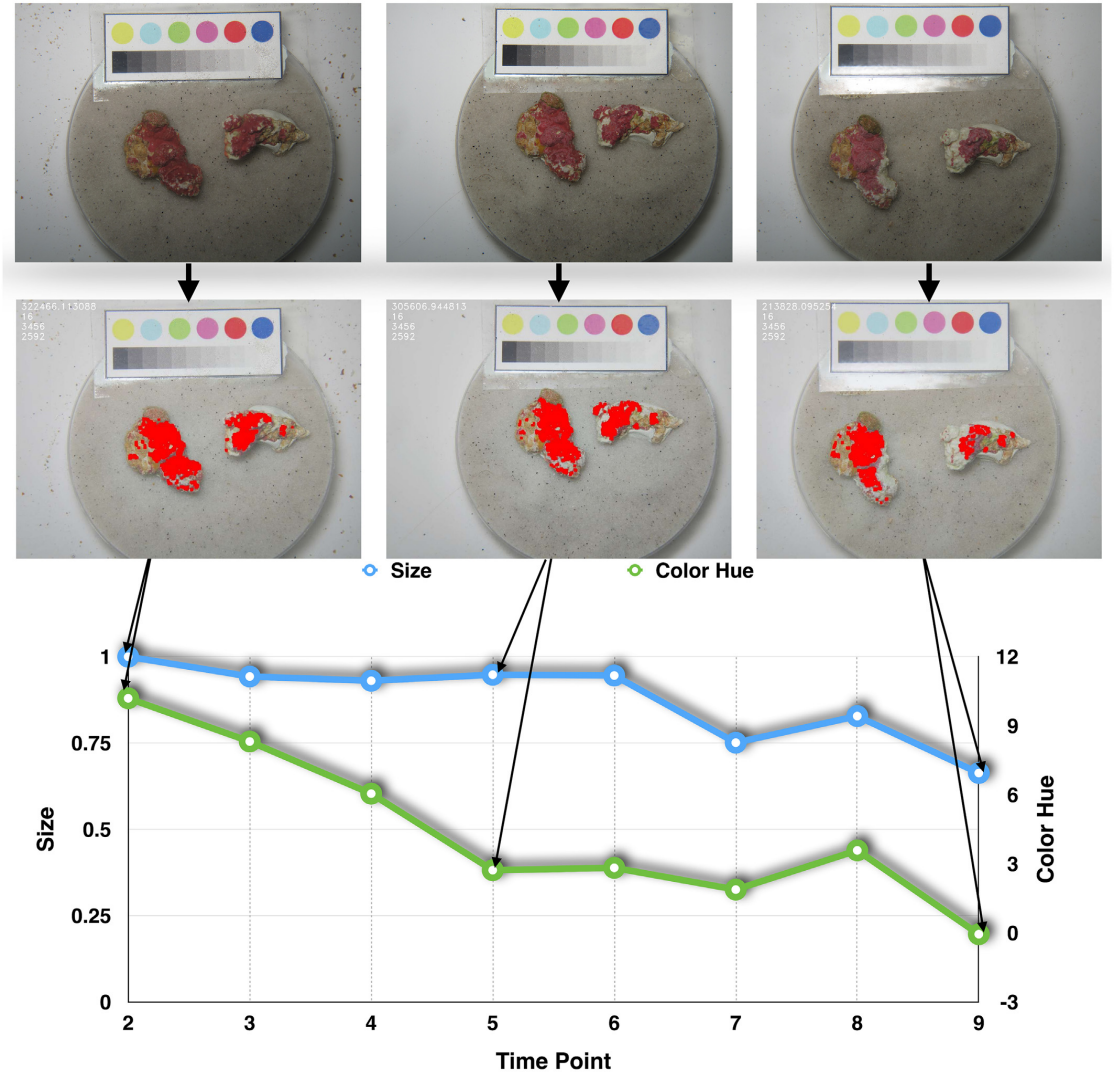
Example of one pair of calcareous algae samples. In the top row the original images recorded at T2, T5 and T9 are shown. In the middle row the segmentation results are highlighted and in the lower row the live calcareous algae relative size ($\hat{A}$) and hue ($\bar{h}$), starting from 10 (slightly orange red) to 0 (red), are presented. The samples were exposed to S=900g (a partially covered sample), $L=6.6\mu \text{mol }{\text{m}}^{-2}\;{\text{s}}^{-1}$ and $F=0.07\text{m}\;{\text{s}}^{-1}$.

To analyze the possible biological impact of sedimentation on calcareous algae, the size and the color of the segmented areas in the images are computed. (Fig 6). The size (*A*) of the live calcareous algae area is measured as the amount of segmented pixels per image *J*^(*n*)^. It is weighted by the ratio of the average color circle distance of circle centers $\bar{d}$ and the average circle center distance $\bar{d_{n}}$ of the color reference plate from the Image *J*^(*n*)^.

$$A=\text{size}\times \frac{\bar{d}}{\bar{d_{n}}}$$ (12)

The relative size $\hat{A}=\frac{A}{A_{0}}$, with *A*_0_ being the live calcareous algae size at the first measurement, is finally used as one variable. The HSV (hue, saturation, value) color space is used for measuring the color. Note that we use a different color space here than the one we used for the segmentation. Averages of hue (averaging is applied with respect that it is a circular quantity) ($\bar{h}$), saturation ($\bar{s}$) and value ($\bar{v}$) of all segmented pixels per image *J*^(*n*)^ are computed of the segmented pixels. These four variables are combined to one measurement for the image *n*:

$$f^{\left(n\right)}={\left(\hat{A},\bar{h},\bar{s},\bar{v}\right)}^{T}$$ (13)

All calcareous algae samples from the same chamber are exposed to the same treatment parameters. On each image two samples were captured. To avoid the need of a colocation of the samples as well as taking into account that all rhodoliths are different, averages of size and color within each chamber are used. The dataset can be found in S1 Table.

Multivariate regression is performed with partial least squares (PLS) [38, 39] on the live calcareous algae area measurements **f**^(*n*)^, using Unscrambler X 10.3 (CAMO Software, Oslo, Norway) to correlate the predictor variables (X matrix) to the measured responses (Y matrix). The previously published data (first experimental series with *M. engelhartii* in [6]) on *P* and *SC* are included in the data matrix for the regression analysis. To match **f**^(*n*)^ also data for *P* and *SC* were averaged chamber wise (six parallels). The final data matrix has 168 rows (21 chambers × 8 time-points) (observations), four columns for the four predictor variables (L, F, S and T), and six columns for the response variables ($\hat{A},\bar{h},\bar{s},\bar{v},P,SC$). Prior to the multivariate data analysis, the data are mean centered and scaled to unit variance. The models were evaluated with respect to explained variance, and goodness of fit and prediction, the latter obtained after cross validation [40, 41].

In addition to the PLS regression, a traditional statistical analysis was conducted by performing ANOVA, MANOVA and pairwise correlation analysis. These results are presented in the supplementary (Table A, Table B, Table C in S2 Text and Fig A, B, C in S3 Text). In the following we will concentrate on the results of the PLS regression as it takes all variables into consideration simultaneously and gives a better understanding of the relative importance and the significance of all variables.

## Results

### Pre-Processing

Fluctuations in sample illumination are a well-known problem in computational image analysis and the majority of the *N* = 630 images *I*^(*n*)^ (*n* = 1, …, *N*) suffered at least partially from it. The procedures described above compensated this effect (Fig 3(B)).

In 15% of the images used in the analysis, the reference color circles could not be detected automatically. In these images the circles were marked manually or only a subset of detected circles was used for the color correction. Although reflections were avoided on the calcareous algae samples, serious reflection problems were observed on the color reference plates in some of the images. The images from T0 and T1 had to be excluded from further analysis because of this problem.

Visual inspection showed that the channel-wise gamma correction produced robust results. The pre-processing stages described above could be computed all together in about three minutes per image.

### Segmentation

The segmentation results were reviewed by comparing the human expert labels with the segmented areas of all images using standard measures for image analysis accuracy assessment like recall (or sensitivity), precision (or positive predictive value) and false positive rate (FPR). The recall is the percentage of all pixels labeled “live” or “stressed” *p*^(+)^ that have been segmented correctly as live calcareous algae by the algorithm.

$$\text{recall}=\frac{\left|\left\{p^{\left(+\right)}\right\}\cap^{\text{​}}\left\{p^{\left(s\right)}\right\}\right|}{\left|\left\{p^{\left(+\right)}\right\}\right|}$$ (14)

The precision is the percentage of all pixels segmented as live calcareous algae (*p*^(*s*)^) that have been segmented correctly by the algorithm.

$$\text{precision}=\frac{\left|\left\{p^{\left(+\right)}\right\}\cap^{\text{​}}\left\{p^{\left(s\right)}\right\}\right|}{\left|\left\{p^{\left(s\right)}\right\}\right|}$$ (15)

From the segmentation results we computed a recall value of 0.89 and a precision of 0.03.

Since the manually labeling did not attempt to measure the precise live calcareous algae extent in each image, also the “dead” and “bare substratum” labels were used to review the segmentation results. The false positive rate (FPR) was used to measure the number of pixels that were segmented to be “live” or “stressed” *p*^(*s*)^ but were labeled “dead” or “bare substratum” *p*^(−)^.

$$\text{FPR}=\frac{\left|\left\{p^{\left(-\right)}\right\}\cap^{\text{​}}\left\{p^{\left(s\right)}\right\}\right|}{\left|\left\{p^{\left(-\right)}\right\}\right|}$$ (16)

**Fig 7:**
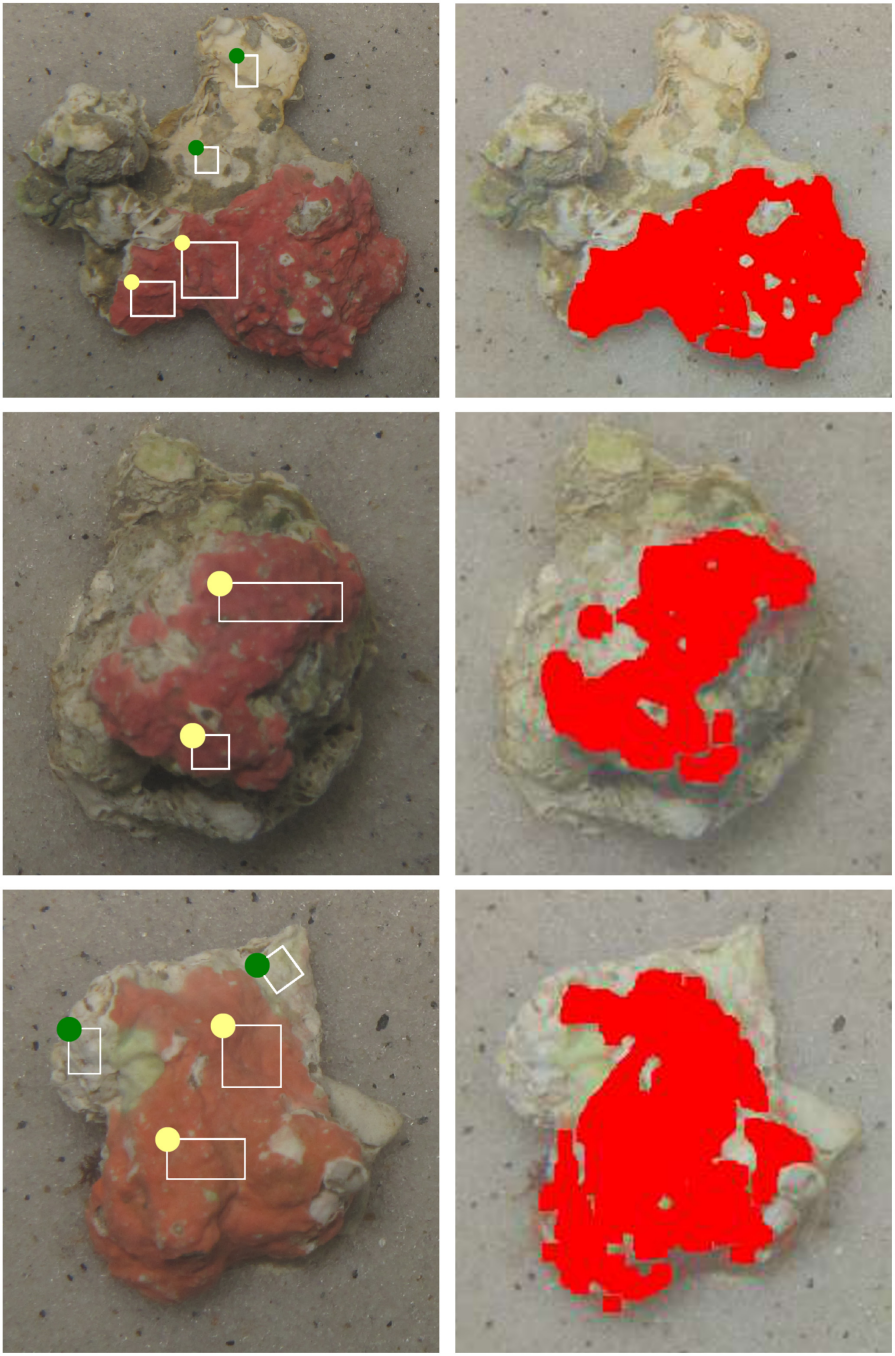
Examples of manually labeled rhodoliths (left) and the corresponding segmented rhodoliths (right). White rectangles with a green dot mark “dead” calcareous algae labeled regions and rectangles with a yellow dot mark “stressed” calcareous algae labeled regions. The pixel regions of “live” or “stressed” calcareous algae are marked red.

The FPR was very low compared to the recall. Looking at the false positive rates in detail it should be noted that the FPR for the “bare substratum” is higher than the one of the “dead” labels (Table 2). Segmentation results are also displayed in Fig 7.

**Table 2:** Results for the evaluation of the segmentation.

Recall	Prec.	FPR		
		“dead“∪ “bare”	“dead”	“bare”
0.89	0.03	0.02	0.02	0.05

The very low values for precision were expected, since a realistic quantification of the FP is not possible in our dataset (see text for details). The low FPR though indicates that the segmentation generates low FP in the “dead” or “bare substratum” labeled regions.

### Multivariate Data Analysis

**Fig 8:**
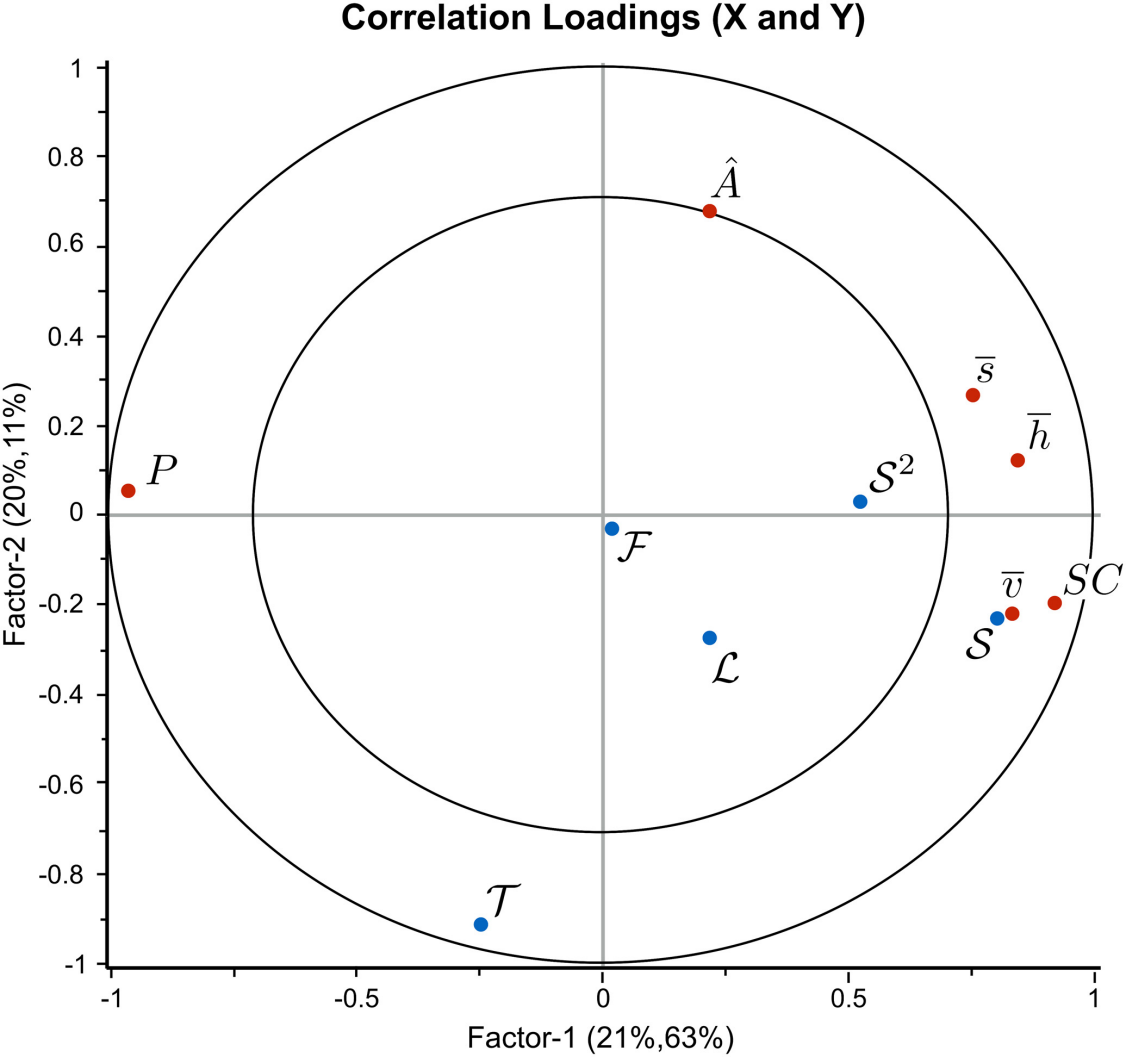
The correlation loading plot obtained after PLS-regression. It was computed with light (L), flow rate (F), amount of sediment (S, $S^{2}$), and time (T) as predictors (blue dots), and HSV-variables ($\bar{h},\bar{s},\bar{v}$), relative size ($\hat{A}$), photosynthetic efficiency (*P*) and sediment coverage (*SC*) as responses (red dots). The outer circle represents 100% explained variance and the inner circle 50% explained variance for the different variables.

The best PLS regression model was obtained after expansion with one square term ($S^{2}$) in the polynomial describing the relationship between predictors (L, F, S and T) and responses ($\hat{A},\bar{h},\bar{s},\bar{v},P,SC$). One significant outlier was identified and removed from the data set, as it appeared that the segmentation failed in the corresponding image (no “live” or “stressed” calcareous algae were detected). Fig 8 shows the correlation loading plot obtained from the PLS-regression. The outer circle represents 100% explained variance and the inner circle 50% explained variance for the different variables.

*SC* and *P*, were both positively and negatively (respectively) correlated with amount of sediment (S) with excellent goodness of fit and prediction. Also the HSV-variables ($\bar{h},\bar{s},\bar{v}$) extracted with our approach, were correlated with S. Goodness of fit and prediction, the latter obtained after cross validation, are summarized in Table 3. Furthermore, there were inter-correlations between the response variables: the HSV-variables were inter-correlated with *SC*, and inversely correlated with *P*. The predictor parameters F and L had low influence on the measured responses. Time (*T*) showed no correlation with *P*, *SC*, or HSV, but was well explained and inversely correlated with relative size ($\hat{A}$). This implies that there are two major directions in the data reflected in the loading plot. The first (horizontal) PLS-component describes S and measured *P*, *SC* and the HSV-variables. The second PLS-component (vertical) describes *T* and $\hat{A}$, which are not correlated with the other variables shown in the loading plot.

**Table 3:** Goodness of fit and prediction of the PLS-regression.

*R*^2^	$\bar{h}$	$\bar{s}$	$\bar{v}$	$\hat{A}$	*P*	*SC*
fit	0.73	0.64	0.76	0.52	0.93	0.93
pred.	0.72	0.62	0.74	0.50	0.93	0.93

Goodness of fit and prediction for the response variables as function of the four predictor variables L, F, S and T.

**Fig 9:**
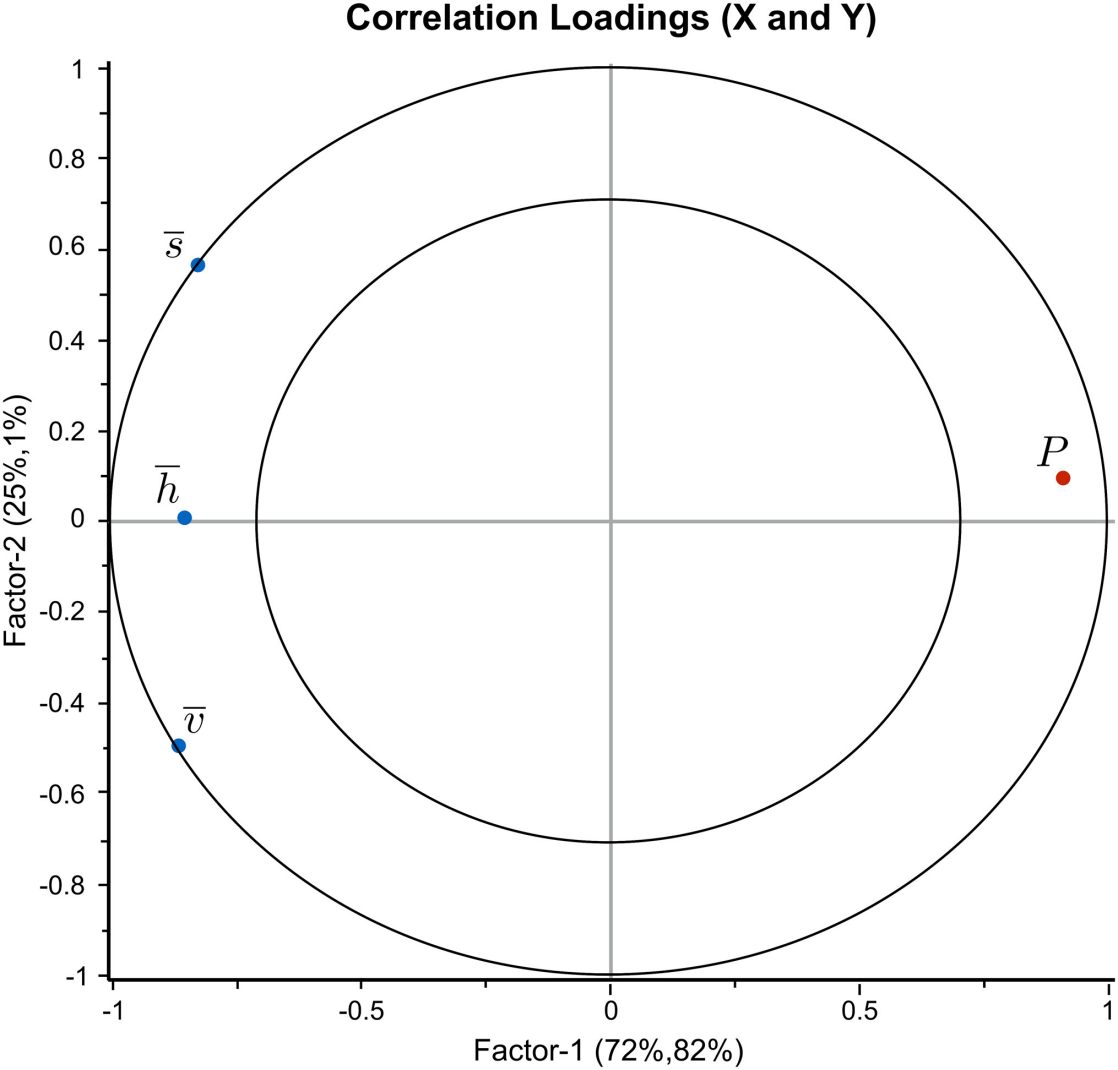
The correlation loading plot for the photosynthetic efficiency (*P*). It was obtained after PLS-regression with the HSV-variables ($\bar{h},\bar{s},\bar{v}$) as predictors (blue dots), and photosynthetic efficiency (*P*) as response (red dot). The outer circle represents 100% explained variance and the inner circle 50% explained variance for the different variables. The plot showed that the predictor variables are highly correlated to the response variable. A prediction of the photosynthetic efficiency using the HSV-values is therefore possible for our experiment.

The apparent inverse correlation between photosynthetic efficiency (*P*) and the HSV-variables suggests an alternative PLS-regression with HSV-variables as predictors and *P* as response. Fig 9 shows the resulting loading plot obtained after PLS-regression with linear relations. Goodness of fit and prediction were both 0.82, enabling prediction of photosynthetic efficiency from HSV-values.

## Discussion

The use of BIIGLE enabled a flexible and swift exchange of data between the researchers involved in this interdisciplinary study, independent of their geographical location.

The selected strategy for the manual labeling in this study was to apply an “example based” labeling approach, rather than to fully outline the different calcareous algae regions. The rationale behind the chosen strategy was that color changes were expected to be time and/or treatment related and that the use of a “example based” annotation approach (rectangles and points) would balance the time spent on labeling and the amount of training data needed. Due to this approach, no representative ground truth for the negative class, i.e. pixel regions that show no calcareous algae, existed. In principle the negative class could have been constructed by a random selection from the unlabeled regions of the images. However this random selection might have introduced some false-negatives to the negative class. This motivated us to apply an unsupervised learning approach. The prototype label mapping in the H^2^SOM followed a conservative strategy to classify only 80% of the positive training data as “live”/“stressed” calcareous algae. Despite of this strategy, our segmentation result showed a high recall of 0.89. Based on the selected “example based” manual labeling strategy the precision was low, as we were expecting. The rationale behind the chosen strategy was that color changes were expected to be time and/or treatment related and that the use of a “example based” annotation approach (rectangles and points) would balance the time spent on labeling and the amount of training data needed. A posterior visual inspection of the results showed that the selected approach produced many false positives that actually turned out to be true positives. Computing the precision as defined in Eq 15 was therefore not a reasonable assessment for the accuracy of our segmentation system (Fig 7). The FPR scores should be low, as an indication of the effect, that almost none of the pixels labeled as “dead” or “bare substratum” were segmented into “live” or “stressed” calcareous algae regions. The FPR for the “bare substratum” is noticeable, but was interpreted as still low and does not seem to have a significant effect on the evaluated colors.

**Fig 10:**
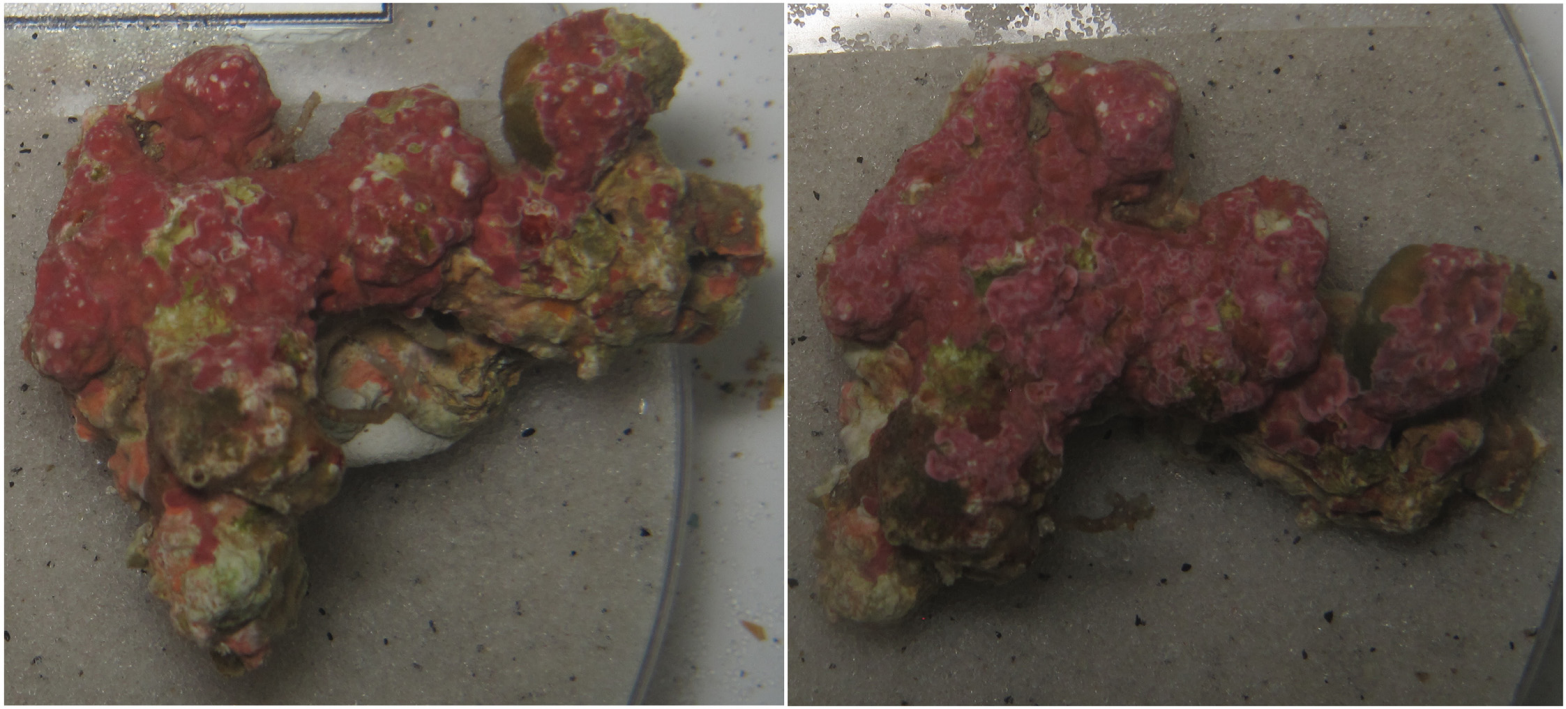
The relative position of the sample can have an effect on the segmentation result. Both images are displaying the same calcareous algae sample, but were recorded at different time points during the experiment. The calcareous algae sample was rotated around an axis not perpendicular to the image plane.

The PLS regression showed that the photosynthetic efficiency is correlated (*R*^2^ = 0.82) to the HSV-values, implying that it is possible to predict the photosynthetic efficiency from the color (Fig 9). The segmented images indicate that the area of live calcareous algae decreases with time during the exposure study. However, the correlation of *P*, *SC* and the color ($\bar{h},\bar{s},\bar{v}$) with time was low, as goodness of fit and prediction are 0.39 and 0.35, respectively (Fig 8). Only the relative size ($\hat{A}$) seemed to be associated with time although the correlation was relatively weak. According to Table 3 goodness of fit and prediction were 0.5 for $\hat{A}$ as a function of all predictor variables, including time. Performing linear regression with $\hat{A}$ as a function of time alone gave a correlation coefficient of *R*^2^ = 0.4. The latter low correlation could have been caused by rotations of rhodoliths around an axis not perpendicular to the image plane between the different time points of the images (Fig 10).

The results of the pre-processing steps show that the illumination fluctuations could be reduced significantly. Furthermore, the reference plate enabled us to achieve color constancy and to correct camera zooming for all images. Although we were able to design a pre-processing pipeline to enhance the image quality and to compensate feature shifts caused by the experimental set up, some experimental steps can still be improved. For instance, in most of the excluded images, the color reference plate showed strong reflections due to a suboptimal material surface and the angle between camera and reference plate. Hence, the circle detection failed or the gamma correction produced wrong corrections if the reflection influenced too many circles. The strong reflections that appeared in the color reference plate may be avoided by selecting a material that gives minimal reflections. Also using an imaging setup where the location and angle of the reference plate are fixed and optimized to avoid strong reflections will improve the image quality. Furthermore using a camera with manual settings enables the use of a fixed white balance that reduces possible fluctuations in color. The necessity to apply illumination correction could maybe be avoided using an optimized stable light setup system with artificial light sources only. Reference plate and sample should be illuminated evenly throughout the whole image. By experience even in laboratory studies these conditions are often hard to achieve throughout the whole experiment. The presented semi-automated software solution can be used to evaluate the images even if not recorded under the optimized conditions, as shown here.

At this state the whole automated segmentation progress takes about 4 minutes for each image, where most of the time is spent on the pre-processing stage. As shown by [31] a significant saving of processing time is achievable by code optimizations and the use general-purpose computing on graphics processing units (GPGPU). A similar acceleration can be expected for our approach, as no code optimization and no GPGPU has been applied for this study.

In comparison to this laboratory study, the analysis of underwater images recorded in the field is even more challenging for the issues discussed above as the levels of the inherent optical properties (IOP) will affect the image quality. Various concentrations of phytoplankton (Chl a), colored dissolved organic matter (cDOM) and total suspended matter (TSM) will alter the contrast, sharpness and colors of objects due to absorption and/or scattering of light [42, 43]. In shallow waters, (0–20 m depth) reflection from the seafloor and varying light conditions related to the cloudiness and flickering effects (focusing/defocusing due to wave effects) would add even more challenges that should be considered [44]. Therefore, it must be expected that image (pre-)processing will be needed when operating under field conditions. As both the calcareous algae and sediments at the Peregrino field moves quite extensively (unpublished results), the monitoring should focus on measurements of possible color change of the segmented (visual) calcareous algae present in the image over time.

Furthermore, we believe that this methodological approach has the potential to be applied to studies with other species where color change is a stress indicator, e.g. tropical corals [7]. However, as individual species have species specific features (color, structure, shape, etc.) and their surroundings differ, species specific adjustment for computational analysis may be required.

## Conclusion

This pilot study has shown that impact of sedimentation on calcareous algae samples can be detected by the presented computational approach for automatic size and color measurement from photos.

In addition to the previously published correlation between photosynthetic efficiency and sediment coverage [6], the present multivariate regression showed correlations between time and calcareous algae size, as well as correlations between fluorescence and calcareous algae color. Furthermore, we have shown that the use of an unsupervised learning algorithm is a promising attempt to create a classifier out of a sparsely labeled dataset. The chosen approach of an automatic assignment of the H^2^SOM prototypes was successful. The results of this pilot study will enable researchers to conduct more studies in the future with different parameterizations and samplings to improve calcareous algae stress models.

The observed correlation between photosynthetic efficiency and the HSV-values is highly interesting for field studies, since photosynthetic efficiency measurements are impractical to perform manually in deeper waters. Color measurement in images has therefore the potential to play an important role in future field calcareous algae monitoring systems.

## Supporting Information

S1 Text (PDF)

S2 Text (PDF)

S3 Text (PDF)

S1 Table (CSV)
