# Supplementary material for: Computational Visual Stress Level Analysis of Calcareous Algae Exposed to Sedimentation
Source: PLoS One. 2016 Jun 10;11(6):e0157329. doi: 10.1371/journal.pone.0157329 (PMC4902238; doi:10.1371/journal.pone.0157329)
Supplement: S1 Text — (PDF) [file pone.0157329.s001.pdf]

S1 Text

Parameter Tuning. Different feature types and different training settings were used and tested for training H^2SOMs. The features were varied regarding the histogram binning (4, 8, or 16 bins per color channel) or regarding the median RGB values (3 dimensional) (Eq. 1), extracted on a 5×5 or 9×9 pixel neighborhood.

$$\mathbf{x}^{(i)} = \begin{pmatrix} \mathrm{bin}_0^{(\mathrm{blue})} \\ \vdots \\ \mathrm{bin}_{B-1}^{(\mathrm{blue})} \\ \mathrm{bin}_0^{(\mathrm{green})} \\ \vdots \\ \mathrm{bin}_{B-1}^{(\mathrm{green})} \\ \mathrm{bin}_0^{(\mathrm{red})} \\ \vdots \\ \mathrm{bin}_{B-1}^{(\mathrm{red})} \end{pmatrix}, B = \{4, 8, 16\} \text{ or } \mathbf{x}^{(i)} = \begin{pmatrix} \underset{(x,y)\in\eta^{(i)}}{\mathrm{median}} \left(p_{(x,y),\mathrm{red}}\right) \\ \underset{(x,y)\in\eta^{(i)}}{\mathrm{median}} \left(p_{(x,y),\mathrm{green}}\right) \\ \underset{(x,y)\in\eta^{(i)}}{\mathrm{median}} \left(p_{(x,y),\mathrm{blue}}\right) \end{pmatrix} \quad (1)$$

Also different random sub-selections (1%, 2%, 5%, 10%) of images and random sub-selection of the amount of feature vectors $\mathbf{x}^{(i)}$ (50%, 12.5%, 6.25%) were tested as well. The expert labels "alive" and "stressed" from BIIGLE were used to determine the best H^2SOM training set using the previously defined *label-likeness* s_j. The "label-likeness" $s_j^{(h)}$ was computed for each training set h. The ten highest $s_j^{(h)}$ of each set h are summed up and the set with this highest aggregated value is determined to by the best one.
