# Supplementary material for: Computational Visual Stress Level Analysis of Calcareous Algae Exposed to Sedimentation
Source: PLoS One. 2016 Jun 10;11(6):e0157329. doi: 10.1371/journal.pone.0157329 (PMC4902238; doi:10.1371/journal.pone.0157329)
Supplement: S2 Text — (PDF) [file pone.0157329.s002.pdf]

S2 Text

Statistical analysis. PLS regression was applied for a comprehensive statistical evaluation and visualization of the relationships between the design variables and the six responses, and is illustrated in Figures 8 and 9 in the manuscript with correlation loadings plots. Also more traditional statistical analysis, i.e. several ANOVA's (Table A) and a MANOVA (Table B) have been used to analyze the data generated from the experimental designs with orthogonal factors and multiple responses. In addition, descriptive statistics with pairwise regression have been computed (Table C). The latter illustrates the importance of the variables although the coefficients are based on pairwise regression. It should be emphasized that the p-values do not reflect the relative importance of the design variables on the responses. As there are a high number of degrees of freedom (168 observations), even minor and unimportant effects may become significant. On the other hand, the regression coefficients from pairwise regression are much lower than the correlation coefficients obtained by PLS-regression taking all variables into consideration, simply because the data are multivariate. Thus, the correlation loadings plots based on PLS regression taking all variables into consideration give a better understanding of the relative importance and the significance of the variables. Furthermore, the PLS-regression models are critically validated using cross validation (Table 3 in manuscript).

Table A. Results of pairwise ANOVA for the experiment data is shown.

	$\hat{A}$	$\overline{h}$	$\overline{s}$	$\overline{v}$	P	SC
$\mathcal{F}$	0.514	0.0672	0.549	0.875	0.329	0.292
$\mathcal{L}$	< **0.001**	0.0261	0.411	0.285	0.0223	0.268
$\mathcal{S}$	0.779	< **0.001**	< **0.001**	< **0.001**	< **0.001**	< **0.001**
Time	< **0.001**	**0.001**	< **0.001**	0.825	0.0556	0.963

Pairwise ANOVA p-values are displayed. Significant p-values are highlighted.

Table B. Results of MANOVA for the experiment data is shown.

Design variables	p-value
$\mathcal{F}$	< **0.001**
$\mathcal{L}$	< **0.001**
$\mathcal{S}$	< **0.001**
Time	< **0.001**
$\mathcal{F} * \mathcal{L}$	< **0.001**
$\mathcal{F} * \mathcal{S}$	0.006
$\mathcal{L} * \mathcal{S}$	< **0.001**
$\mathcal{F} *$ Time	0.518
$\mathcal{L} *$ Time	0.002
$\mathcal{S} *$ Time	< **0.001**
$\mathcal{F} * \mathcal{L} * \mathcal{S}$	< **0.001**
$\mathcal{F} * \mathcal{L} *$ Time	0.618
$\mathcal{F} * \mathcal{S} *$ Time	0.131
$\mathcal{L} * \mathcal{S} *$ Time	0.015
$\mathcal{F} * \mathcal{L} * \mathcal{S} *$ Time	0.275

MANOVA p-values are displayed. Values are computed using Wilks' Lambda. Significant p-values are highlighted.

Table C. Pairwise variable correlation analysis for the experiment data is presented.

	$\hat{A}$	$\overline{h}$	$\overline{s}$	$\overline{v}$	P	SC
$\mathcal{F}$	0.003	0.020	0.002	0.000	0.006	0.007
$\mathcal{L}$	0.085	0.029	0.004	0.007	0.031	0.007
$\mathcal{S}$	0.000	0.391	0.215	0.464	**0.642**	**0.780**
Time	0.418	0.087	0.196	0.000	0.022	0.000
$\hat{A}$	1	0.062	0.255	0.006	0.028	0.004
$\overline{h}$	0.062	1	0.387	0.364	**0.646**	**0.627**
$\overline{s}$	0.255	0.387	1	0.247	0.381	0.319
$\overline{v}$	0.006	0.364	0.247	1	0.544	0.555
P	0.028	**0.646**	0.381	**0.544**	1	**0.845**
SC	0.004	**0.627**	0.319	**0.555**	**0.845**	1
$\mathcal{S}^2$	0.011	0.250	0.139	0.132	0.272	0.148

Coefficients of determination R^2 are displayed. Values > 0.5 are highlighted.
