# Supplementary material for: Computational Visual Stress Level Analysis of Calcareous Algae Exposed to Sedimentation
Source: PLoS One. 2016 Jun 10;11(6):e0157329. doi: 10.1371/journal.pone.0157329 (PMC4902238; doi:10.1371/journal.pone.0157329)

## S3 Text

**Figure A. Mean values of hue ($\bar{h}$) and photosynthetic efficiency ($P$) grouped by added amount of sediment ($\mathcal{S}$) are presented to illustrate the impact of sedimentation.** In the upper graph mean values of $\bar{h}$ grouped by amount of added sediment ($\mathcal{S}$) over time with standard deviation as error bars are displayed, showing the correlation between $\bar{h}$ and $\mathcal{S}$. In the lower graph mean values of $P$ grouped by $\mathcal{S}$ over time with standard deviation as error bars are displayed, showing the correlation between $P$ and $\mathcal{S}$.

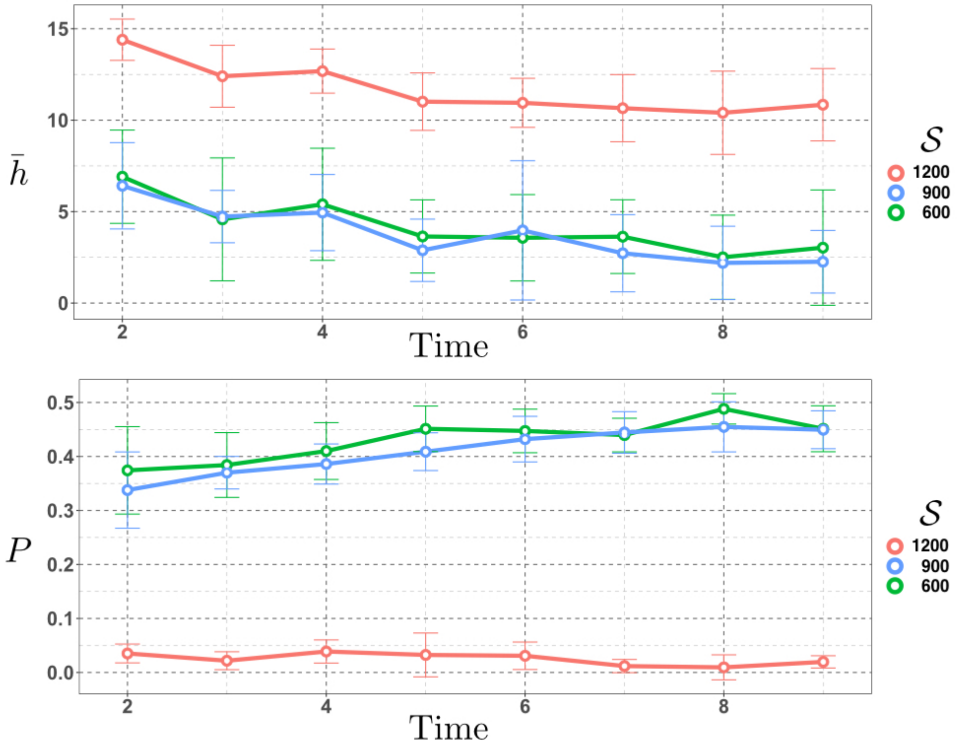

**Figure B. The Data plot for mean hue ($\bar{h}$) against photosynthetic efficiency ($P$).** Color code is used to distinguish between different amounts of added sediment ($\mathcal{S}$.)

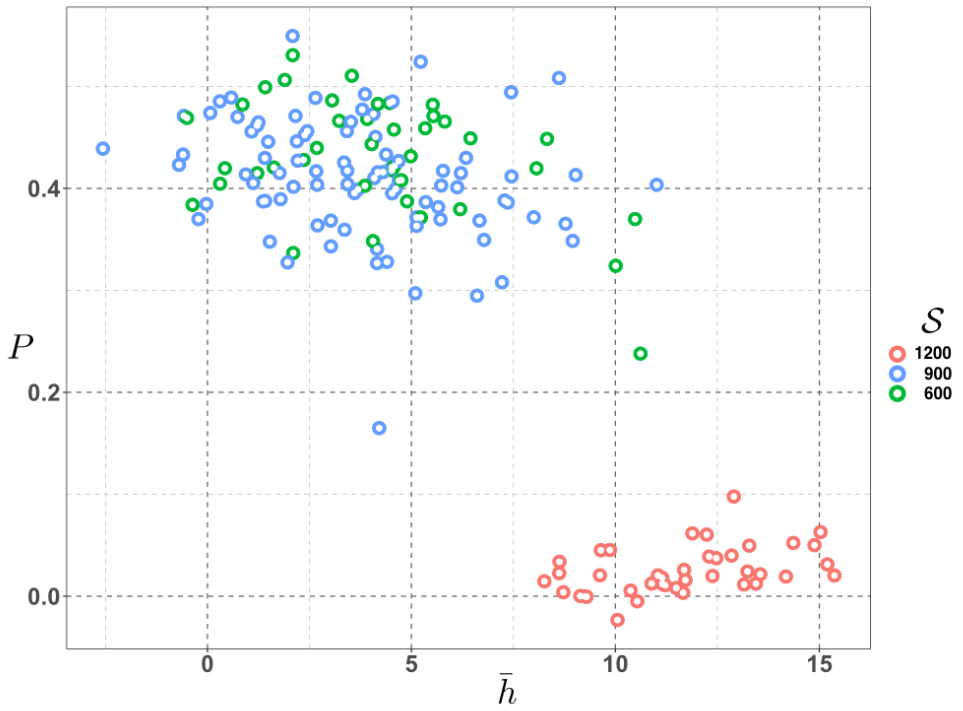

**Figure C. Data plot for mean values of relative size $\hat{A}$ against Time.** Error bars are showing standard deviation.

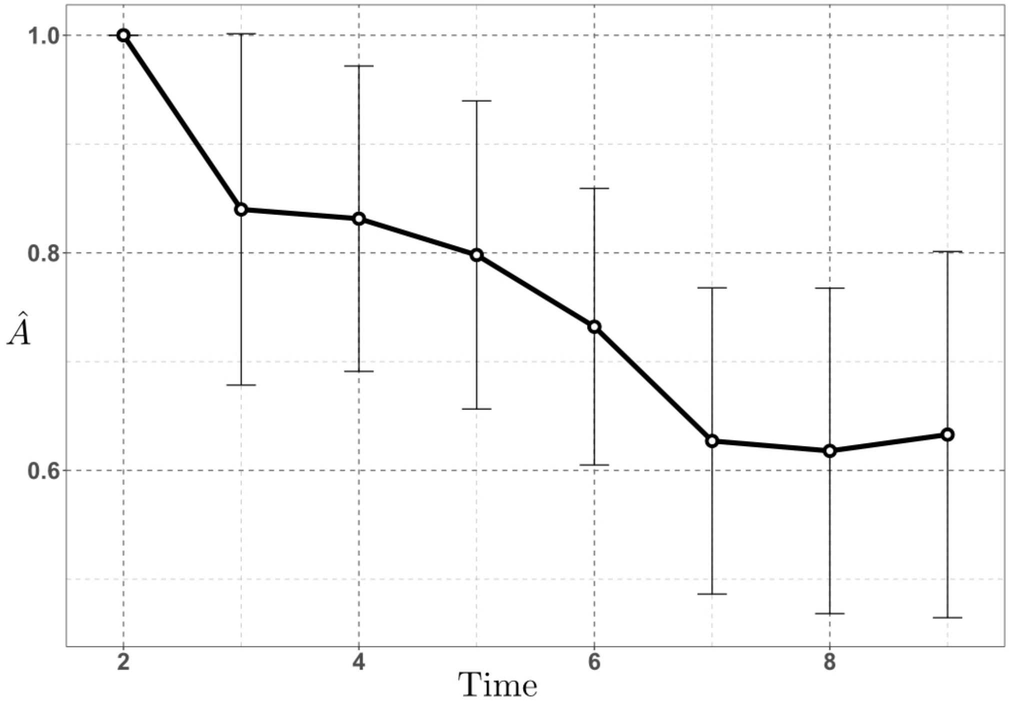

Supplement: S3 Text — (PDF) [file pone.0157329.s003.pdf]
